# Sibling Adjustment and Sibling Relationships Associated with Clusters of Needs in Children with Autism: A Novel Methodological Approach

**DOI:** 10.1007/s10803-020-04854-0

**Published:** 2021-01-13

**Authors:** Louise Rixon, Richard P. Hastings, Hanna Kovshoff, Tom Bailey

**Affiliations:** 1 Centre for Educational Development Appraisal and Research (CEDAR), University of Warwick, Coventry, CV4 8UW UK; 2 The Centre for Innovation in Mental Health (CiMH), School of Psychology, University of Southampton, Southampton, UK; 3 Department of Psychiatry, School of Clinical Sciences at Monash Health, Monash University, Clayton, VIC 3800 Australia

**Keywords:** Autism, Siblings, Cluster analysis, Sibling adjustment, Sibling relationships

## Abstract

We tested a novel methodological approach to examine associations between characteristics of autistic children and outcomes for siblings. Cluster analysis was used to define five groups of children with autism (n = 168) based on autism symptoms, adaptive behavior, pro-social behavior, and behavior problems. Primary and secondary parent carers, and siblings themselves, reported on sibling relationship quality and psychological adjustment. Siblings of autistic children with a mild symptom profile, high levels of adaptive skills, but high internalizing and externalizing problems had the highest level of these problems themselves and more conflict in their relationship. Siblings of autistic children with the most complex support needs (adaptive skills deficits, severe autism symptoms) reported lower warmth relationships but not elevated internalizing and externalizing problems.

Recent review and meta-analytic evidence suggest that siblings of children with autism (hereafter referred to as autism siblings) are more likely than other siblings to experience behavioral and emotional problems (Meadan et al. 2010; Pisula and Ziegart-Sadowska 2015; Shivers et al. 2018; Thomas et al. 2016). There is also evidence that sibling relationships may be less positive when one of the children in a sibling dyad has autism (Brewton et al. 2012; Petalas et al. 2012a, b; Ross and Cuskelly 2009). As sibling relationships may support positive development for all children (e.g. McHale et al. 2016; Moss et al. 2019), this is an area of concern both for siblings and for children with autism.

Although these broad findings of putative negative impact on sibling well-being and sibling relationship quality have been replicated, outcomes in the literature on autism siblings show considerable variability. Not all autism siblings have poor outcomes, and not all sibling relationships are characterized by negative or difficult experiences (Meadan et al 2010; Petalas et al. 2012a, b; Shivers et al 2018). For example, Tomeny et al. (2012) studied the risk of poor social, emotional and behavioral adjustment of 42 (17 males and 25 females aged 6–18) neurotypical autism siblings and a control group of neurotypical sibling pairs. They found that levels of externalizing behavior problems, internalizing and social problems of autism siblings did not differ significantly from the siblings in the control group. Evidence of the positive nature of the sibling relationship also exists in the literature. Diener et al. (2015) studied boys with autism and their typically developing sisters. The typically developing sisters reported how much they enjoyed spending time with their brothers, the pleasure they gained from helping them, and the importance they placed on the relationship.

The individual characteristics of the child with autism such as behavior problems, the severity of autism symptoms, and the severity of any other co-morbid conditions may have an exacerbating, or attenuating, effect on functioning of typically developing siblings and/or on sibling relationships. In particular, behavior problems of the child with autism are often found to be a predictor of poorer sibling outcomes and less positive sibling relationship quality (Hastings 2007; Macks and Reeve 2007; Walton 2016; Walton and Ingersoll 2015). In terms of the severity of autism symptoms (e.g. the quality of social relationships, social communication, social understanding, and repetitive and restrictive behaviours) of the child with autism, this has sometimes been found to be a predictor of sibling outcomes and sometimes not (Jones et al 2019; Sacrey et al. 2018).

Researchers examining the characteristics of children with autism that may affect sibling outcomes and sibling relationships have also examined the independent direct contribution of, for example, behavior problems, adaptive behavior, and autism symptoms (Bontinck et al. 2018; Tomeny et al. 2017). However, other research in autism has conceptualized the different dimensions of the needs of the child with autism in a more holistic manner. We use the general term “needs” to refer to autism symptoms, prosocial and adaptive skills, and behavior and emotional problems of the child with autism in the current paper; and not to necessarily imply directly associated needs for support or services. In particular, researchers have examined whether the needs of children with autism are associated in meaningful ways such that there may be sub-groups or phenotypes of autism defined by need. This issue has been addressed mainly using cluster analysis and related approaches. For example, Klopper et al. (2017) studied 61 children (5–14 years) with autism without intellectual disability (ID) to investigate clinically meaningful subgroups. Using IQ, language, pragmatic communication, and behavior Klopper et al. were able to establish that children with autism without ID could be divided into two clear “moderate” and “severe” subgroups characterized by different levels of social impairment. Similarly, Bitsika et al. (2018) studied 147 young males (6–18 years) with autism and found a two-cluster solution of high and low severity of autistic symptomatology. Researchers have also subtyped autism using restricted and repetitive behaviours (e.g., Zheng et al. 2019), and reported that three subgroups were evident: low, medium, and high levels of restricted and repetitive behaviors.

In the present research, we hypothesized that to examine how and to what degree the characteristics of children with autism may be associated with their siblings’ well-being and the quality of the sibling relationship, a multi-dimensional approach is likely to be informative. The cluster analysis literature shows that the needs and characteristics of children with autism co-occur in meaningful sub-groupings. Therefore, in principle, the characteristics of children with autism may not act independently but rather in concert in exerting any putative effects on siblings. To examine this line of argument, in the current study we first developed cluster groupings within a sample of children with autism (using multiple indicators of needs/characteristics) and then compared sibling outcomes and sibling relationships across the resulting groups. In keeping with a call to examine data from different reporters about siblings (Kovshoff et al. 2017), we gathered data about sibling adjustment and sibling relationships from mothers, fathers, and siblings themselves; then comparing these data across the needs-based cluster groupings.

## Method

### Participants

#### Primary Parents/Carers

Parents from 168 families took part in this research. The majority of the primary parent/carer respondents were female (92.8%), being either the mother or primary female carer; fathers or male carers equated to 5.4% of the total. Primary parent carers were aged between 26 and 64 years, with a mean age of 42.17 years (SD = 7.12). 163 (97.0%) of the parents described themselves as White British, White Irish or Other White, with the remaining five parents describing themselves either as White and Black Caribbean, Asian/Asian British-Pakistani, Black/Black British Caribbean, Asian/Asian British Indian or another Asian background. With regards to marital status, 142 primary parent/carers (84.5%) were married and living with their spouse and 26 (15.5%) were divorced, separated, single or widowed and not living with their partner. 78 (46.4%), were educated to a postgraduate or undergraduate degree level, and 64% of parental primary carers worked outside of the home; 30 full-time, and 77 part-time.

#### Secondary Parents/Carers

Secondary parents/carers from 129 of the 168 families took part in this research with the majority being the father or male carer (96.9%) and the remainder being mothers or secondary female carers. Secondary carers were aged between 27 and 63 years, with a mean age of 43.71 years (SD = 5.60). 123 described themselves as White British, White Irish or Other White with the remaining two describing themselves as either Any Other Mixed Background or Any Other Black background. In terms of marital status, 98.4% of the secondary parents/carers were married and living with their spouse/partner was and 0.8% were divorced, separated, single or widowed and not living with their partner. 43 (43.4%) were educated to an undergraduate degree, or equivalent, level, and 94.6% of secondary parents/carers worked outside of the home; 113 full-time, and 9 part-time. To reduce participant burden and encourage participation, only one sibling measure was completed by secondary carers.

#### Children with Autism

The children with autism from the 168 families were 137 boys (81.5%) and 31 girls (18.5%), aged between five and 17 years, with a mean age of 10.43 years (SD = 2.85). Based on primary caregiver report (no independent diagnostic information was available), 102 (60.7%) children had a diagnosis of autism, 64 (38.1%) had a diagnosis of Asperger’s syndrome, and 2 (1.2%) children were diagnosed with PDD-NOS (pervasive developmental disorder not otherwise specified). 60 children (35.7%) had an additional condition, including one child with Down Syndrome, 18 with ADHD or hyperactivity, and 41 with another, unspecified, additional diagnosis. 25 (14.8%) of the children attended a mainstream school with no support, 90 (53.6%) children attended mainstream school with support, 9 (5.4%) children attended a specialist unit within a mainstream school, 35 (20.8%) children attended a special school, and 9 (5.4%) children attended other educational services.

#### Autism Siblings

The 146 autism sibling participants (not all families had a sibling within the required age range of 8–17 years) were chosen by the primary caregiver as the child closest in age to the child with autism, and the siblings were typically developing (according to parental report, they did not have a psychiatric or disability diagnosis). The rated siblings ranged in age from 5 to 17 years (mean age = 10.60 years, SD = 3.74). 77 were brothers, and 69 were sisters. 73 (50.0%) were younger than the child with autism, 68 (46.6%) were older, and 5 (3.4%) were twins. 57 (39%) of the siblings attended the same school as their brother or sister, and 86 attended a different school (missing data n = 3).

### Measures

#### Child with Autism Measures

The Strengths and Difficulties Questionnaire (Goodman 1997) is a 25-item screening instrument for child behavior and emotional problems and prosocial behavior. The primary parents/carers were asked to complete the 4–16 years SDQ version about the child with autism. In the current research, scores for pro-social behavior (5 items), and the combined domains of externalizing (conduct and hyperactivity, 10 items) and internalizing problems (emotional and peer problems, 10 items) were used. Parents/carers were asked to complete questions about their child’s behavior over the preceding six-month period. Examples of questions asked of the parents/carers about their autistic child include: internalizing ‘does your child have many worries or often seem worried?’; externalizing ‘does your child often lose their temper?’; pro-social ‘does your child often volunteer to help others (parents, teachers, children)?’. The use of the internalizing and externalizing problems scores has been shown to provide the clearest and most consistent evidence of convergent and discriminant validity for the SDQ across informants (Goodman et al. 2010). Internal consistency (Cronbach’s alpha) in the current study for the children with autism was 0.77 for prosocial behavior, 0.69 for internalizing problems, and 0.74 for externalizing problems.

The Vineland Adaptive Behavior Rating Scales 2nd Edition (Sparrow et al. 2005) was completed with primary parent/carers to assess the adaptive skills of their child with autism. Using a structured interview format, the VABS gathers information on the day-to-day activities necessary to take care of oneself and to assess the development of personal independence and socialization with others. For the purposes of this study the communication, daily living skills, and socialization standard scores were used; motor skills being primarily used as a measure for children up to five years of age or with individuals with a recognized deficit in mobility.

The Social Communication Questionnaire (SCQ: Rutter et al. 2003) is an autism-screening instrument, consisting of 40 yes/no questions. The primary parent/carer completed the questionnaire using the Current Form of the measure. This records the child’s symptoms within the preceding three-month period and is therefore not a diagnostic tool but measures the current severity of symptoms. The three domains of restricted, repetitive and stereotyped patterns of behavior; communication; and reciprocal social interaction were used in the current analyses. Examples of the eight restricted, repetitive and stereotyped questions include: verbal rituals ‘does she/he ever say the same thing over and over in exactly the same way or insist that you say the same thing over and over again?’ and unusual sensory interests, “Does she/he ever seem to be *unusually* interested in the sight, feel, sound, taste or smell of things or people?’. Communications questions (13 in total) include: conversation, ‘Do you have a to and fro “conversation” with her/him that involves takings turns or building on what you have said?’ and imaginative play, ‘Does she/he play any pretend or make-believe games?’. Reciprocal social interaction (15 questions) include: interest in children, ‘does she/he seem interested in other children of approximately the same age whom she/he does not know?’. The Kuder Richardson Coefficient for the individual domains in the current study was 0.74 for Reciprocal social interaction; 0.61 for restricted, repetitive and stereotyped behavior; and 0.60 for Communication.

#### Sibling Measures

Siblings themselves completed the Harter Self Perception Profile and the global self-worth score was used to provide an overview of siblings’ global concept of self, constituting a general perception of self, rather than personal self-judgement of ability or sense of adequacy (Harter 2012). A higher total represents a higher global self-worth of the individual completing the questionnaire. Statements include, ‘some kids are very happy being the way they are BUT other kids wish they were different’ and ‘some kids are happy with themselves as a person BUT other kids are often not happy with themselves’. Using a scale of ‘really true to me’ to ‘sort of true to me’ the procedure is to score each item on a four-point scale from 1 to 4. A score of 1 indicates the lowest global self-worth and a score of 4, the highest. Cronbach’s alpha coefficient in the current sample for the global self-worth scale was 0.93.

Siblings and primary carers both completed the Sibling Relationship Questionnaire (SRQ) to assess the dimensions of the sibling relationship (Buhrmester and Furman 1990). The SRQ is a 39-item questionnaire measuring 16 dimensions of the sibling relationship. The questionnaire consists of four sub-scales: warmth/closeness; relative status/power; conflict; and rivalry with items rated using a five-point Likert scale of 1 hardly ever to 5 extremely much. The parent-report version asks the parent to rate how well a particular characteristic describes the relationship between two siblings, and the self-report version focuses on how autism siblings in this case perceive their sibling relationship. A higher score on each of the sub-scales or factors indicates a greater level of the specified quality in the sibling relationship. Cronbach’s alpha in the current study for sibling self-reports were: warmth/closeness 0.92; rivalry 0.87; conflict 0.88; and relative status/power 0.68. For primary carers, Cronbach’s alphas were: warmth/closeness 0.90; rivalry 0.77; conflict 0.86; and relative status/power 0.63.

Both the primary and secondary parent/carer completed the SDQ about the autism sibling, and the autism sibling completed a self-report version of the SDQ suitable for young people where the sibling was aged 11–16 years. Cronbach’s alpha for the autism siblings for the SDQ in the current study were: primary carer pro-social 0.81; Internalizing 0.85 and externalizing 0.85; secondary carer pro-social 0.78; internalizing 0.76. and externalizing 0.81; and self-reported SDQ pro-social 0.79; internalizing 0.73 and externalizing 0.81.

### Procedure

#### Sample

The data were collected as part of a larger research study of families of children and young people with autism (Petalas et al. 2012a, b). Following ethical approval, invitations were given to a national autism charity to distribute to their members through their local and national groups. As these groups distributed this study material through direct contact, advertising and mailing lists, the total number of families contacted at this stage is unknown. A total of 305 families made contact with the research team and met the criteria of having a primary caregiver in the home and a child with autism between the ages of 5 and 17 years. The families were mailed questionnaire packs including separate packs for primary and secondary carers, and siblings if they were between 8 and 17 years of age. Participants were requested to complete questionnaires independently of each other. Participants from a total of 215 families completed questionnaires within the time frame of the research. Of these 215 families, 168 (78.1%) were included in the current study because they had at least one autism sibling.

#### Creating the Cluster Groupings

Data were analyzed using IBM SPSS statistics version 24. A hierarchical agglomerative cluster analysis was performed on the data to initially determine the presence of sub-groups of children with autism. This stepwise clustering method combines observations into subgroups, the agglomerative method placing each case into an individual cluster. The most similar clusters merge together to create new clusters, continuing until they create a single group (Hair and Black 2000). The advantage of this type of analysis is its unbiased nature, allowing a reduction in data in an objective manner based on descriptive and multivariate techniques (Cholemkery et al. 2016).

The nine variables included within the initial clustering model were from those measures assessing different dimensions of needs of children with autism as reported by the primary parental caregivers (to maximise sample size): SDQ internalizing, externalizing and pro-social behavior; VABS communication, socialization, and daily living skills; and SCQ reciprocal social, communication, and restricted behavior scores. All scores were z transformed before being entered into the analysis since different scoring ranges and scales are used in each measure. The squared Euclidean distance was used as a measurement of proximity, and Ward’s method to merge the clusters. A dendrogram was examined and suggested four potentially viable cluster solutions (ranging from four to seven clusters). A series of K-means cluster analyses were then applied extracting between four and seven clusters. The five-cluster and six-cluster solutions were retained as they led to more coherent groupings of needs.

Comparison of the cluster means (for the variables used to cluster the autistic children), in both the five and six cluster group solution, was conducted using analyses of variance (ANOVA) to determine which of the solutions gave the clearest classification of the data. These ANOVA results, combined with cross tabulation of membership of clusters from the two different solutions, suggested that the five-cluster group solution was the most comprehensive and concise of the solutions, demonstrating clear delineation of the groups whilst retaining the individual detail within the clusters. To further examine the five and six cluster solutions, demographic data for age and gender were analyzed using chi-squared tests, cross tabulation, and ANOVA to highlight potential further differences between the cluster groups. Balancing all available data, the five cluster-group solution provided both the best homogeneity within, and the greatest separation between, the subgroups. The cluster group statistics for the five-cluster solution were compared across the clusters for the nine variables used to derive the clusters. Mean scores for primary carer ratings of their autistic children according to these nine variables are presented in Table 1.

**Table 1:** Descriptive statistics—5 cluster membership

	Cluster 1	Cluster 2	Cluster 3	Cluster 4	Cluster 5	p (sig)**	F ***
	(n = 37)	(n = 43)	(n = 40)	(n = 20)	(n = 28)		
	Mean (SD)	Mean (SD)	Mean (SD)	Mean (SD)	Mean (SD)		
SCQ reciprocal social	6.80 (3.08)	10.29 (3.03)	7.41 (2.76)	4.47 (1.52)	2.78 (1.72)	< .001	38.82
SCQ communication	5.96 (2.30)	9.08 (2.02)	7.99 (1.93)	7.15 (2.16)	4.85 (1.62)	< .001	23.63
SCQ restricted behavior	4.06 (1.77)	6.52 (1.21)	5.42 (1.37)	6.50 (1.10)	2.92 (1.60)	< .001	36.01
VABS communication	71.75 (9.21)	57.06 (9.00)	74.55 (9.03)	82.15 (15.80)	83.71 (11.92)	< .001	35.19
VABS daily living skills	64.18 (7.80)	54.60 (9.21)	70.45 (8.87)	76.25 (10.94)	80.46 (14.09)	< .001	34.96
VABS socialization	60.94 (7.37)	50.90 (7.13)	66.40 (8.09)	77.25 (9.32)	75.35 (8.29)	< .001	60.43
SDQ pro social	1.83 (1.46)	1.79 (1.83)	3.58 (2.20)	4.55 (1.43)	5.67 (2.03)	< .001	26.87
SDQ internalizing	9.97 (3.13)	11.70 (3.20)	14.61 (2.51)	8.61 (2.73)	7.46 (3.43)	< .001	28.59
SDQ externalizing	10.83 (3.56)	10.90 (3.03)	13.46 (2.50)	10.50 (2.78)	7.25 (3.15)	< .001	17.58

*SCQ* social communication questionnaire, *VABS* Vineland adaptive behavior scales, *SDQ* strengths and difficulties questionnaire

**p (sig) values < 0.0001 across all variables

***F values refer to analysis of variance (ANOVA) that compared cluster group scores

The autistic children in Cluster 1 (n = 37) had an overall profile of relatively high levels of internalizing difficulties, but fewer autism symptoms. They had a mean age of 10.57 years and were 81% male. This cluster was characterized by elevated externalizing behaviors alongside increased anxiety and emotional adjustment (internalizing problems).

Cluster 2 (n = 43) represents a group of autistic children with multiple complex needs. Their profile includes elevated autism symptoms and self-help needs potentially requiring a high level of support. They showed significant problems across of all of the primary parental caregiver ratings, scoring the highest of the five clusters on the SCQ for reciprocal social, communication, and restricted repetitive behavior domains. VABS subdomains, including communication, daily living skills and socialization, showed that this group had significant learning and adaptive behavior needs. Cluster 2 was the oldest of the groups with a mean age of 11.33 years, and was 84% male.

Cluster 3 (n = 40) can be described as displaying moderate autism symptom severity and moderate adaptive skill needs This group had a relatively high internalizing and externalizing problems profile. Children in this group had a mean age of 10.16 years, and 78% of them were male.

Cluster 4 (n = 20) is the smallest of the groups, with relatively fewer autism symptoms and restrictive and repetitive behaviors that were similar to the multiple complex needs group in Cluster 2. They showed relatively high independence and self-help skills, though also had elevated levels of externalizing problems, and a low prosocial SDQ score. Cluster 4 represented the youngest of the groups (M = 9.36 years) and was 90% male.

Finally, the autistic children represented by cluster 5 (n = 28) showed the highest levels of adaptive skills across all of the VABS domains. They displayed fewer autism behaviors and low levels of behavioral and emotional problems. The group was the second youngest with a mean age of 10.01 years and was 79% male.

## Results

Following the development of groups of autistic children informed by cluster analysis, the main analysis focusing on the study research question, explored group differences between the clusters in terms of their siblings’ outcomes using a series of one-way ANOVAs. Post-hoc Tukey HSD tests were used to identify the cluster groups differing from each other on a sibling outcome when the overall ANOVA showed a statistically significant difference across the cluster groupings. Mean scores for outcomes as rated by primary carer, secondary carer, and autism siblings are presented in Tables 2 and 3. There were a number of statistically significant cluster group differences as reported by the primary carers, and for the siblings’ self-reported ratings of outcomes. No cluster group differences were apparent for sibling outcomes as reported by secondary carers.

**Table 2:** Sibling subscale group scores—primary carer (PC) and secondary carer (SC) ratings

	Cluster 1		Cluster 2		Cluster 3		Cluster 4		Cluster 5				
	Mean (SD)	(n =)	Mean (SD)	(n =)	Mean (SD)	(n =)	Mean (SD)	(n =)	Mean (SD)	(n =)	F	p (sig)	Group differences
Primary carer rating													
PC SDQ prosocial	7.64 (2.32)	28	8.05 (1.80)	39	7.85 (2.55)	34	8.28 (1.85)	14	8.63 (1.59)	22	0.808	.522	
PC SDQ internalizing	5.00 (4.27)	28	3.17 (3.25)	39	5.76 (4.53)	34	4.50 (4.05)	14	2.63 (2.68)	22	3.343	.012	5 < 3
PC SDQ externalizing	6.50 (5.41)	28	4.98 (3.93)	39	6.64 (3.78)	34	5.42 (3.99)	14	3.22 (2.99)	22	2.887	.025	5 < 3
PC SRQ warmth/closeness	2.04 (0.50)	27	2.04 (0.59)	38	2.27 (0.79)	34	2.74 (0.58)	14	2.75 (0.42)	22	7.741	< .001	1, 2 < 4, 5
PC SRQ relative status/power	0.17 (0.70)	28	0.54 (0.62)	36	− 0.05 (0.61)	34	0.16 (0.61)	14	0.40 (0.58)	22	4.471	.002	3 < 2
PC SRQ conflict	3.36 (1.18)	28	2.41 (0.96)	38	3.72 (0.67)	34	2.65 (0.67)	14	2.63 (0.87)	22	11.752	< .001	2, 5, 4 < 1, 3
PC SRQ rivalry	0.51 (0.42)	27	0.37 (0.37)	38	0.51 (0.44)	32	0.38 (0.39)	14	0.29 (0.34)	21	1.444	.223	
Secondary carer rating													
SC SDQ prosocial	7.68 (2.07)	22	7.84 (2.52)	33	8.18 (2.08)	22	7.45 (1.50)	11	8.35 (2.17)	17	0.438	.781	
SC SDQ internalizing	4.04 (3.52)	22	3.36 (3.19)	33	4.38 (3.21)	21	3.72 (3.74)	11	3.82 (2.67)	17	0.347	.846	
SC SDQ externalizing	6.22 (4.16)	22	4.87 (4.15)	33	5.09 (2.72)	22	5.63 (4.43)	11	4.17 (3.22)	17	0.814	.519	

*SDQ* strengths and difficulties questionnaire; *SRQ* sibling relationship questionnaire; *Harter* self-perception profile

**Table 3:** Sibling subscale group scores—self scored ratings

	Cluster 1		Cluster 2		Cluster 3		Cluster 4		Cluster 5				
	Mean (SD)	(n =)	Mean (SD)	(n =)	Mean (SD)	(n =)	Mean (SD)	(n =)	Mean (SD)	(n =)	F	p (sig)	Group differences
SDQ prosocial	7.09 (2.38)	11	7.29 (1.96)	17	8.27 (2.10)	11	8.00 (1.41)	4	8.66 (1.15)	12	1.467	.226	
SDQ internalizing	5.22 (3.34)	11	4.05 (2.92)	17	5.54 (3.53)	11	7.25 (5.90)	4	5.00 (2.33)	12	0.908	.466	
SDQ externalizing	6.54 (3.67)	11	6.54 (4.43)	17	7.27 (3.74)	11	6.18 (3.57)	4	3.41 (2.74)	12	1.870	.130	
SRQ warmth/closeness	3.06 (0.82)	17	2.86 (0.77)	26	2.53 (1.03)	17	2.77 (1.06)	8	3.36 (0.58)	15	2.140	0.084	
SRQ relative status/power	0.26 (0.66)	17	0.34 (0.56)	26	0.22 (0.52)	17	0.03 (0.45)	8	0.26 (0.32)	16	0.567	.687	
SRQ conflict	2.81 (1.12)	17	2.36 (0.99)	26	3.66 (0.96)	17	2.56 (1.25)	8	3.02 (0.88)	16	4.425	.003	2, 4 < 3
SRQ rivalry	0.44 (0.32)	15	0.44 (0.48)	26	0.68 (0.64)	16	0.38 (0.49)	8	0.40 (0.37)	15	0.989	.419	
Harter global self worth	3.22 (0.51)	18	3.02 (0.66)	29	3.09 (0.75)	22	3.02 (0.96)	8	3.34 (0.48)	16	0.778	.542	

*SDQ* strengths and difficulties questionnaire; *SRQ* sibling relationship questionnaire; *Harter* self-perception profile

For SDQ scores, there were a number of overall group differences for siblings as reported by the primary parent/carer including on internalizing F(4,132) = 3.343, p = 0.012 and externalizing F(4,132) = 2.8873, p = 0.025 problems. Primary carer ratings of internalizing problems in siblings whose autistic brothers and sisters were in cluster 3 (M = 5.76, SD = 4.53) were higher than for siblings who had autistic brothers and sisters in cluster 5 (M = 2.63, SD = 2.68). This group difference was mirrored in the primary parent/carers’ ratings of externalizing problems: siblings of autistic children in cluster 3(M = 6.64, SD = 3.78) had higher scores than the siblings of the children in cluster 5 (M = 3.22, SD = 2.99).

There were also overall cluster group differences for primary carer reported sibling relationship ratings of warmth and closeness F(4,130) = 7.741, p = < 0.001, relative status/power F(4,129) = 4.471, p = 0.002, and conflict F(4,131) = 11.752, p = < 0.001; and for sibling self-reported conflict F(4,79) = 4.425, p = 0.003. For the SRQ warmth/closeness domain the primary carers of the siblings whose autistic brothers and sisters were in clusters 4 (M = 2.74, SD = 0.58) and 5 (M = 2.75, SD = 0.42) reported that there was more warmth/closeness in the relationship than for the siblings with brothers and sisters in cluster 1 (M = 2.04, SD = 0.50) and 2 (M = 2.04, SD = 0.59). Primary carers reported that the siblings of brothers and sisters in cluster 3 (M = -0.05, SD = 0.61) demonstrated lower status and power in the sibling relationship than the siblings whose brothers and sisters in cluster 2 (M = 0.54, SD = 0.62).

In terms of the SRQ conflict score, primary carers of siblings whose autistic brothers and sisters were in cluster groups 1 (M = 3.36, SD = 1.18) and 3 (M = 3.72, SD = 0.67) reported more conflict in the sibling relationship than for siblings whose brothers and sisters were in cluster groups 2 (M = 2.41, SD = 0.96), 5 (M = 2.63, SD = 0.87) and 4 (M = 2.65, SD = 0.67). Siblings themselves whose autistic brothers and sisters were in cluster group 3 (M = 3.66, SD = 0.96) also reported more conflict in their relationship than siblings whose brothers and sisters were in cluster groups 2 (M = 2.36, SD 0.99) and 4 (M = 2.56, SD = 1.25).

## Discussion

We hypothesized that multi-dimensionally defined needs of children with autism (i.e., clusters, or sub-types) across a range of indices would be associated with siblings’ psychological adjustment and sibling relationship quality. From a sample of children with autism across the 5–17 years age range, we successfully derived five meaningful clusters with varying levels of associated strengths and needs. These groups ranged from a complex needs group to a group of autistic children who may be perceived as more able on some measures (e.g., with regards to autisms symptoms or independence skills) but with high levels of internalizing and externalizing problems.

Whilst it was important for the methodology of the current study to identify clusters of children with autism, the primary aim was not to identify autism cluster groups per se. Previous research has also identified clusters by autism symptomology (e.g. Bitsika et al. 2018; Zheng et al. 2019), social impairments (e.g. Klopper et al. 2017), and levels of ability or functioning (e.g. Stevens et al. 2000). Whilst we were also able to identify similar subgroups in the present study, relative to previous research identifying autism group clusters, we used a wider range of variables to identify the different groups of children with autism.

The results of the group comparisons of sibling outcomes and relationship quality suggested that it is unhelpful to hold a simple expectation that increased severity of problems associated with the child with autism necessarily leads to poorer sibling outcomes. The siblings of brothers and sisters with likely the most complex support needs in cluster 2 (high autism scores and relatively poorer adaptive skills) displayed lower internalizing and externalizing problems compared to siblings of children in cluster groups 1, 3 and 4. Autism siblings of children in Cluster 2 also benefitted from a sibling relationship that was relatively free from conflict, as reported by primary carers and self-reports, compared to siblings of children in cluster groups 1 and 3; although they did also have lower levels of warmth and closeness in the sibling relationship according to the primary carer reports. In addition, siblings of children with higher levels of adaptive skills, fewer autistic traits, and lower levels of internalizing and externalizing problems (cluster 5) also had relatively more positive outcomes. This group of siblings had the highest parent/carer reported warmth and closeness scores, low parent/carer scores for sibling conflict and the lowest reported levels of internalizing and externalizing behaviors as reported by parent/carers. The siblings who were reported as having the highest levels of problems themselves were those of the autistic children with a relatively mild autistic profile but higher internalizing and externalizing problems and lower levels of prosocial skills (cluster 3). Siblings of children in this cluster had more internalizing and externalizing problems than siblings of children in cluster 5, who displayed the highest levels of adaptive skills, fewer autism behaviors and low levels of internalizing and externalizing problems.

In addition to the possibility that the patterns of needs of the children with autism are directly related to the sibling outcome group differences found here, the group differences may have been driven indirectly. Specifically, parental stress might also vary by cluster group and this could have an effect across the family unit, including on siblings (Meadan et al. 2010). Family systems perspectives might also suggest that family sub-system stresses may mediate the impact of clusters of need in children with autism on siblings. For example, martial stress (which may also vary with the needs of children with autism) has also been found to be an important correlate of sibling relationship quality in families of children with autism, with siblings reporting that they felt they directed more negative and fewer positive behaviors toward their brother or sister with autism when marital stress was high (Wood-Rivers and Stoneman 2003). Langley et al. (2017) reported a negative association between child behavior problems and relationship satisfaction in parents of children with autism within a study exploring relationship satisfaction, highlighting the importance of maintaining parental boundaries. These boundaries may become tested if the child with autism also has behavior problems.

A further explanation for the associations found in the current study, especially in relation to siblings’ internalizing and externalizing problems, might be shared genetic vulnerability. For example, high risk siblings (those with an older brother or sister with a diagnosis of ASD) have been shown to have elevated levels of autistic traits and more social communication impairments, lower cognitive abilities and more internalizing problems than typically developing children (Georgiades et al. 2013; Messinger et al. 2013; Pisula et al. 2015). It has also been suggested that the presence of the broad autism phenotype in the parents can be associated with the severity of autism in the child (Sasson et al. 2013), with the lowest severity being found in the parental pairs with no presence of a broad autism phenotype feature.

The use of a multi-dimensional approach using multiple respondents gives a comprehensive view of sibling outcomes. By utilizing both primary and secondary carer reports alongside those of the sibling themselves we were able to have a better understanding of how the siblings not only feel about their relationship with their brother or sister with autism, but also how they feel about themselves. Even the different patterns of findings related to different respondents can in itself present an opportunity to understand individual siblings’ functioning (Rankin et al. 2017). These differences are also evident within the sibling relationship, especially for sibling relationship quality, siblings may spend a considerable amount of time together in the absence of their parents or carers, and so may be reporting on quite different experiences (Cook and Kenny 2004; Orsmond and Fulford 2018). It is also possible that siblings are comparing themselves with their autistic brother or sister and may, therefore, view themselves more favorably in self-report measures (Macks and Reeve 2007). Understanding the outcomes for autism siblings may also give a better opportunity for not only parents, but also health, education and social services, to have a more cohesive understanding of the support these children may need. This could be of particular relevance to the families of siblings in the highest risk groups where their needs might include strategies to cope with their brother or sister’s externalizing behaviors, learning strategies for communication with their brother or sister, and parental support to allow for more time spent with the sibling.

Several limitations should be considered in this study. First, the range of variables that could affect siblings and the measures available to evaluate sibling adjustment and the quality of relationships is extensive in previous research (Shivers et al. 2018), and further research could explore other outcomes. For example, sibling coping styles, adjustment, and functioning in school and home environments and stress responses could be examined. Second, the data were collected as part of a research study of families of children and young people with autism (Petalas et al. 2012a, b) and the recruitment method included a number of biases of unknown impact. In particular, the participants were drawn from a self-selecting group of families who were approached by a national autism charity and it is not known how these families differed from non-responders and whether they were representative of families of children with autism. The families who did respond were from a relatively high-income bracket which could suggest a group with greater resources than other families. Third, smaller sample sizes on secondary parents/carers’ reports and sibling self-reports meant that it was not possible to have a full understanding of all the families in the study. Lastly, the children with autism within the original study were included on the criteria that they had a clinical diagnosis of autism. However, autism diagnosis was self-reported by the primary caregiver and not verified by a clinician.

The current research focused on one sibling in the family. However, autism families may include multiple siblings (Stanford et al. 2020). Therefore, researchers in future should consider ways to address within-family variability by understanding the relationships and behavioral differences of multiple siblings within the same family. This could provide a deeper understanding of the impact of autism on both individuals and the whole-family dynamic. Additionally, understanding how the children with autism view their neurotypical siblings and their relationships with them could enhance understanding of family dynamics even further. The perspective of children and young people with autism about their siblings has been studied rarely (Petalas et al. 2009), and obtaining the perspectives of children with autism would be an important future research focus, although gathering perspectives from children with autism who also have considerable communication difficulties would be a challenge.

Overall, the current study has shown the feasibility of a new methodological approach to exploring whether or how the characteristics of children with autism may be related to sibling outcomes. The results are of interest directly, but replication and extension of this approach is needed.
